# Co‐Producing and Evaluating a Culturally Inclusive Dementia Education Initiative: A Multimethod Study Protocol

**DOI:** 10.1111/hex.70307

**Published:** 2025-05-27

**Authors:** Gabriela Caballero, Ann Dadich, Michelle DiGiacomo, Nicky Morrison, Charles Okafor, Joyce Siette, Genevieve Z. Steiner‐Lim, Dementia Friends Unite Stakeholders, Diana Karamacoska

**Affiliations:** ^1^ NICM Health Research Institute Western Sydney University Sydney Australia; ^2^ School of Business Western Sydney University Sydney Australia; ^3^ Translational Health Research Institute (THRI) Western Sydney University Sydney Australia; ^4^ Improving Palliative, Aged and Chronic Care through Clinical Research and Translation (IMPACCT), Faculty of Health University of Technology Sydney Sydney Australia; ^5^ Urban Transformations Research Centre Western Sydney University Sydney Australia; ^6^ Centre for Health Services Research, Faculty of Medicine The University of Queensland Brisbane Australia; ^7^ The MARCS Institute for Brain, Behaviour, and Development Western Sydney University Sydney Australia

**Keywords:** Alzheimer's disease, brain health, co‐design, cognitive impairment, health literacy, multicultural

## Abstract

**Introduction:**

Limited dementia awareness among culturally and linguistically diverse communities can exacerbate stigma and hinder support for carers and people at risk of or living with dementia. Co‐producing a culturally inclusive dementia education intervention with representative stakeholders can address these knowledge and service gaps. This paper details the protocol for designing and evaluating a co‐produced multilingual dementia education intervention named Dementia Friends Unite. This project aims to improve dementia knowledge, attitudes and supportive practices in a multicultural context.

**Method:**

This project will be conducted in South Western Sydney, Australia, where Arabic, Cantonese, English, Greek, Mandarin and Vietnamese are the most common languages spoken. A multi‐stakeholder collaboration involving representatives from each of these communities was formed to co‐produce the multilingual dementia education intervention. Two studies are planned to explore the co‐production process and evaluate the intervention's impact, guided by implementation science frameworks. Study 1 will examine stakeholder and researcher experiences in co‐production through minuted meetings and responses to the patient and public engagement evaluation tool. Data will be descriptively analysed to identify the barriers and facilitators to co‐production. Study 2 involves evaluating the initiative's impact according to the RE‐AIM framework. Outcome measures include intervention reach and effectiveness in changing participants' knowledge, attitudes and supportive practices through questionnaires (pre‐, post‐ and follow‐up) and interviews; adoption and implementation characteristics through focus groups with stakeholders and facilitators; and maintenance through a cost–benefit analysis.

**Conclusion:**

This project will employ a comprehensive approach to address unmet needs and research gaps in co‐produced dementia education and its implementation in multicultural contexts. It can serve as a blueprint for others seeking to engage culturally diverse populations in community‐based health education and research.

**Patient or Public Contribution:**

A multi‐stakeholder collaboration involving representatives from local government and care services, as well as people with living and caring experiences of dementia, from each of the targeted communities, was formed to co‐produce this initiative. Their involvement spans study design, conduct, interpretation of findings and dissemination.

## 1 Background

Dementia is a neurodegenerative condition characterised by cognitive and functional decline [1]. Approximately 55 million people live with dementia, a number projected to double every 20 years [2]. Despite increasing prevalence, dementia awareness gaps remain prevalent in culturally and linguistically diverse diasporas where dementia is often perceived as taboo, a mental illness or as normative ageing [3]. These misconceptions underpin delayed diagnoses, service underuse and poorer health outcomes [4, 5, 6]. These disparities are reinforced by culturally inappropriate services, resources and service providers' beliefs regarding familial caring practices [7, 8, 9]. Collaborating with communities is essential to deliver interventions that effectively improve dementia literacy, attitudes and help‐seeking efforts [10, 11, 12, 13].

To promote sustainable partnerships and social change, participatory action research methods are recommended for underserved groups, including people living with dementia [14, 15, 16]. Co‐production can address unmet needs by prioritising equal decision‐making by engaging service users (people with dementia and care‐partners) and service providers (e.g., organisations) as co‐researchers throughout a project's life cycle [17, 18, 19]. Unlike traditional knowledge‐to‐action models that often limit service user involvement to consultation roles, co‐production actively engages them as co‐researchers, creating and resulting in more accessible and acceptable interventions [16, 19]. Collaborating with dementia‐related organisations and local governments can further enrich the process by contributing contextual solutions [20, 21]. For example, a dementia awareness initiative designed, delivered and disseminated by academics, people with living and caring experiences, and local government improved attitudes and reduced stigma in regional Australia [20].

Conducting dementia education in multicultural contexts requires strong community involvement to optimise reach, engagement and learning [13]. Key enablers of this include partnering with cultural/community leaders, people impacted by dementia, care‐partners and organisations; providing resources in‐language; and adequately training bilingual workers and staff [11]. To promote knowledge retention, in‐language materials must be tailored to simplify complex dementia‐related medical terms using culturally responsive language and colloquialisms, while being mindful of cultural stigma [6, 22]. Through cultural adaptation, one Australian multilingual dementia prevention animation iteratively negotiated with Arabic, Cantonese, Greek, Hindi, Italian, Mandarin, Spanish, Tamil and Vietnamese community members about scripting, translations, literacy, cultural stigmas and representative imagery [23]. The resulting culturally inclusive animation was presented in‐language and led to improved knowledge about dementia risk in 318 people from 10 language groups [24]. The co‐creation of another online dementia awareness initiative with Arabic, English and Vietnamese communities in Sydney, Australia, similarly explored dementia literacy and stigma to develop culturally sensitive material [3]. Bilingual community workers were trained to deliver the content as their insider knowledge could enhance rapport and bridge the gap between cultural expectations (e.g., caring norms) and mainstream dementia services [22]. While the initiative supported participants' knowledge about dementia causes, impacts and care strategies, co‐creators acknowledged that the online format during the Covid‐19 pandemic limited accessibility and reach [5]. These recent examples of multicultural partnerships for dementia education fostered equitable access to information and resources, highlighting their ecological validity. As evidenced above, targeting multiple subgroups simultaneously addresses common barriers and the development of broadly applicable, inclusive solutions. This approach also promotes collective awareness, resource efficiency, broader reach and referrals to care services [25]. However, it remains unknown if such initiatives can alter stigma over time. Culturally tailoring materials might be more appropriate and necessary within certain groups to overcome more nuanced issues (e.g., spiritual beliefs and systemic or structural determinants of health).

## 2 Aim

The present educational initiative, Dementia Friends Unite, builds on previous efforts to address unmet educational needs within multicultural communities [3, 7, 8]. It employs a comprehensive approach to co‐production research by integrating theory‐informed education with stakeholder collaboration and evaluation guided by an implementation science framework. The aims of this project are to co‐produce and pilot a multilingual dementia education initiative for its impacts on dementia knowledge, attitudes and supportive practices, such as help‐seeking behaviours and person‐centred care, among adults from linguistically diverse backgrounds.

## 3 Methods

### 3.1 Setting

This project is set in South Western Sydney, where an estimated 13,457 people live with dementia [26]. This multicultural region has 45% of the population speaking a language other than English, including Arabic, Cantonese, Greek, Mandarin and Vietnamese [27]. The funding, resourcing and partnerships activated for this project enabled these language groups to be targeted (see Supporting Information 1 for further details).

### 3.2 Stakeholders

This project will be conducted through a multi‐stakeholder collaboration involving the Canterbury‐Bankstown Dementia Alliance, South Western Sydney Dementia Network and a Dementia Advisory Group (see Tables S1 and S2). Stakeholders were invited to participate via intermediaries or directly through ongoing local partnerships established in previous work [5, 8].

### 3.3 Design

Co‐production and implementation science frameworks were selected given their demonstrated benefits [28, 29, 30]. Implementation science will guide the translation of research into practice throughout the project. The four co's model will be used to scaffold collaboration across the co‐planning, co‐design, co‐delivery and co‐evaluation stages [31]. The co‐production of this educational initiative will be explored in one study, while its impact on individuals and organisations will be evaluated in a second study, according to the RE‐AIM framework [32]. RE‐AIM evaluates an intervention's reach, effectiveness, adoption, implementation and maintenance [33, 34, 35]. In this project, a multi‐method single, within‐group, pre‐to‐post, quasi‐experimental intervention design was selected to determine if Dementia Friends Unite improves dementia knowledge, attitudes and supportive practices. The project was approved by the Western Sydney University Human Research Ethics Committee (reference number: H15904).

## 4 Study 1: Co‐Production of a Multilingual Dementia Education Initiative

The first study focuses on the co‐production process using an observational descriptive design to capture the dynamics of stakeholder collaboration and their experiences. The four Co's model was used to operationalise participatory research across co‐planning, co‐design, co‐delivery and co‐evaluation [31]. Adhering to this model offers clearly defined project phases for implementation and stakeholder involvement throughout the project life cycle [31].

### 4.1 Co‐Planning

A series of hybrid meetings will be held with members of the Canterbury‐Bankstown Dementia Alliance and South Western Sydney Dementia Network. Dedicated meetings will be held with the Dementia Advisory Group (see Table S1) to ensure the objectives reflect the specific needs and preferences of people with living and caring experiences of dementia. Stakeholders will be briefed on the research process and co‐production methodology, discuss concerns and contribute to project design. Three key educational themes will be addressed in this initiative: dementia understanding, accessibility and inclusivity strategies. These themes shaped the aims and impact measures described in this protocol. Given the diverse languages targeted (i.e., Arabic, Cantonese, English, Greek, Mandarin and Vietnamese), content will be co‐designed inclusively across all groups as opposed to culturally tailoring each one to maximise cost‐effectiveness and scalability.

### 4.2 Co‐Design

This process will combine stakeholder input with teaching and learning principles from social constructivism [36]. Here, adult learning is viewed as a socio‐cultural process, where knowledge and attitudes are shaped through meaningful interactions [37]. This andragogical approach emphasises interactive learning, reflection and collaboration among peers and facilitators to promote meaningful knowledge construction and retention [37, 38]. A summary of andragogical teaching and learning strategies will be shared with stakeholders to enable informed decision‐making. Implementation‐related topics regarding intervention delivery format, structure, duration, content, locations, promotional avenues, risk assessment and evaluation methods will also be explored to ensure contextual applicability and adoption by stakeholders. The researchers will minute and mediate hybrid ‘world café’ style workshops held in‐person at a university campus and online via Zoom to optimise access. World cafés have been used in dementia interventions to leverage natural conversational dialogue as participants rotate between topics to exchange knowledge, explore issues and innovate solutions [39]. Material revisions will prioritise input from living experience members until 70% consensus among stakeholders is reached. This threshold allows for consensus and the inclusion of diverse perspectives, compared to similar studies with homogeneous stakeholders reaching higher agreement [18, 36]. This iterative process helps the content meet the majority's needs, preferences and sensitivities. The final version of the educational material will be translated into the different languages using an accredited service, and accuracy checks will be conducted by bilingual research team members and stakeholders.

### 4.3 Co‐Delivery

The diffusion of innovations theory will guide implementation by aligning the initiative with organisational priorities, leveraging diverse communication channels and providing practical resources to promote adoption and sustainability [40, 41, 42]. The research team will organise accessible delivery sites with stakeholders through a series of minuted online meetings and emails. Facilitators for intervention delivery will be recruited through stakeholder networks based on their commitment to advocacy, cultural understanding and community connections. The research team will conduct a half‐day training session with facilitators to review educational materials and role‐play activities. The lead researcher, with over 10 years of tertiary and community‐based teaching experience [5], will integrate the key tenets of social constructivism to ensure that facilitators understand their role in promoting active learning among participants [38]. The facilitators will receive comprehensive notes to equip them with the necessary knowledge and resources to deliver the initiative [43].

### 4.4 Co‐Evaluation

Stakeholders will remain engaged during this project's evaluation processes through hybrid meetings. The research team will conduct the analyses and prepare plain language summaries for discussion at stakeholder meetings. Recognising that each stakeholder might offer unique perspectives on the implications for their community or organisation, this collaborative evaluation might improve the interpretation of findings [5]. Dissemination opportunities will include co‐authoring academic and in‐language lay reports regarding project outcomes, implications and future directions. Iterative feedback on drafts will be collected through email correspondence and minuted meetings. The final lay reports will be sent via email or post to all stakeholders and the initiative participants who opt to receive such information.

### 4.5 Co‐Production Assessment and Reporting

A detailed account of co‐production and its evaluation will be documented in a separate academic paper upon project completion. The validated patient and public engagement evaluation tool (PPEET) will be used to monitor stakeholder experiences and co‐production integrity [17, 44]. This survey captures researcher and stakeholder perspectives on project integrity, outcomes, sustainability and equity, an area of crucial but limited understanding in co‐produced dementia education [39]. The PPEET's focus on dyadic stakeholder collaboration is favoured in health‐related interventions compared to traditional satisfaction metrics, which do not capture multi‐perspective experiences [45].

The quantifiable PPEET questions probe participation support, the exchange of perspectives and the impact of engagement. Responses are rated on a five‐point Likert scale, from *strongly disagree* to *strongly agree*. The PPEET also captures responses to open‐ended items and demographic information, such as age, gender, education and employment. Recommended in implementation science, the PPEET will be administered at various co‐production time points to check and adjust practices that equitably support stakeholder participation. Ongoing co‐production evaluations enable prioritisation and the opportunity to adapt methods when stakeholder feedback is incompatible or suggests low engagement, potentially mitigating the declining motivation reported in other studies [45]. The researchers involved in co‐production will, respectively, complete modules A, B and C before co‐design, after co‐delivery and after co‐evaluation, while the stakeholders will complete the participant‐specific module B after each project phase.

The PPEET survey will be hosted on Qualtrics and completed by accessing a QR code or link. Raw, quantifiable data will be exported from Qualtrics to the Statistical Package for the Social Sciences (SPSS) version 29 for analysis. Descriptive statistics will be calculated as this is a novel exploration of the PPEET in a co‐produced dementia education initiative. Likert scale survey ratings will be converted to numerical values from 1 to 5. Descriptive statistics, including means, standard deviations, medians and interquartile ranges, will illustrate response characteristics across various time points. Ratings will be aggregated according to the meta‐criteria of each survey module, such as process integrity and inclusivity, with higher ratings reflecting greater levels of stakeholder engagement [46].

Conventional content analysis will be conducted by a PhD candidate using responses to the open‐ended PPEET items and meeting minutes. This will serve to identify the barriers and facilitators of co‐production. Themes can be constructed from under‐researched topics or data that offer limited richness, where in‐depth qualitative analyses are unwarranted [47]. Following Hsieh and Shannon's [48] approach, an iterative open coding process will inductively identify meaningful phrases and label them with codes. These codes will be grouped into categories based on explicit and observable content, with broader themes capturing the essence of the findings [49]. To minimise researcher bias, positionality and analytical decisions will be reflectively journaled and debriefed with a senior researcher who was not involved in the co‐production process. Open‐ended survey items will be quantified, and the meeting notes will be narratively synthesised to understand the barriers and facilitators of co‐producing with diverse stakeholders in dementia education research.

## 5 Study 2: Impact Evaluation

The second study employs a multimethod design to comprehensively evaluate the initiative's impact among participants and stakeholders based on the RE‐AIM framework [32] and using a single, within‐group pre‐to‐post intervention design. Data will be collected and analysed according to measures of reach and effectiveness (on participants), adoption (by stakeholders), implementation (with facilitators) and maintenance.

### 5.1 Reach on Participants, Recruitment and Sample Size

This educational intervention targets Arabic, Cantonese, English, Greek, Mandarin and Vietnamese speaking adults over 18 years of age using purposive sampling as each of these communities is represented through the stakeholder groups. Recruitment drives will occur via word of mouth, digital and print media outlets, social media platforms (e.g., WhatsApp and WeChat) and stakeholder networks using in‐language promotional materials. Reach will be tracked by the number of trained co‐facilitators engaged to deliver the initiative and the number of registered attendees at each in‐language session. Based on the previous pilot of an online dementia awareness session that reached 63 participants across three language groups [3] and another study that had 22 participants at follow‐up [33], a minimum of 20 participants per language group (*N* = 120) is expected for the current project.

### 5.2 Effectiveness on Participants

A sequential mixed‐methods design will be used to establish how effective the initiative is at modifying participants' dementia knowledge, attitudes and supportive practices [50]. The first measure is a quantitative survey designed to assess dementia knowledge and attitudes, respectively, using the dementia knowledge assessment scale (DKAS) [51] and an Australian dementia diagnosis attitudes scale (DDAS) [52], as favoured by the Canterbury Bankstown Dementia Alliance members. Ten DKAS items probing dementia causes, symptoms, risk factors and management strategies are rated on a four‐point Likert scale (*false, probably false, probably true* and *true*). Thirteen DDAS probe reactions to a hypothetical dementia diagnosis with the statement, ‘If I were diagnosed with dementia…’. The items query feelings, such as humiliation, shame, embarrassment, anxiety and depression; disclosure to family, employers and health insurers; and perceived care from health professionals. Each item is rated on a five‐point Likert scale from *strongly agree* to *strongly disagree*. The survey also captures demographic information including age, gender, languages spoken, country of birth, employment status, highest level of education and whether they or someone they know has a dementia diagnosis. The survey underwent forward and backward translation by independent certified translators with comprehension and accuracy checks by bilingual researchers. Structural validity of the translated scales was established in other studies using factor and reliability analyses with *α* ≥ 0.70 [53, 54, 55].

The combined dementia knowledge and attitudes survey will be administered before the intervention commences (baseline; Time 1), immediately after the intervention concludes (post‐intervention; Time 2) and 12 months later (follow‐up; Time 3). This follow‐up period aligns with the recommended minimum time frame to evaluate dementia‐friendly initiatives [56]. Participants will receive an information sheet and verbal explanations of the study's purpose, risks, benefits and voluntary nature, with implied consent sought through survey completion. To promote survey completion, participants can enter a draw to win one of six $200 gift cards (i.e., one gift card per language group). Bilingual research assistants, with at least an Honours degree, will be trained to administer the surveys by telephone at baseline and follow‐up and in person immediately post‐intervention using printed versions. Completed surveys will be transcribed by bilingual researchers for efficient data management and then exported to SPSS for analysis. Relevant test assumptions for each scale item will be assessed, and the internal consistency of responses to the DKAS and DDAS will be checked using Cronbach's *α* ≥ 0.70 [57]. Pre/post‐intervention changes in DKAS and DDAS outcomes will be assessed by independent samples *t*‐tests on each scale item for normally distributed data. The equivalent Mann–Whitney *U* test will be used for non‐normally distributed data. Outcomes will be considered statistically significant at *p* < 0.05, one‐tailed. Data will be presented as means and standard deviation with medians, mean ranks and interquartile ranges. Survey participation rates are expected to vary across time points [33], so demographic variables will be analysed using the *χ* ^2^ test to identify group differences between time points.

Building on the surveys, semi‐structured interviews about short‐term behavioural changes will be conducted with survey participants who opt for follow‐up. The interview schedule will probe experiences with dementia, motivations for attending, any changes in supportive practices like improved dementia care or help‐seeking, and the barriers and facilitators of knowledge application (see Supporting Information 2 for the full interview schedule). Bilingual research assistants will be trained by an experienced qualitative researcher on interview processes. Interviews will be conducted in‐language, audio‐recorded and transcribed into English for analysis. The interview transcripts will not be shared with the participants for review or feedback.

Interview transcript data will undergo qualitative content analysis via NVivo version 14 [58]. Data‐driven patterns and themes will be interpreted to enable contextual understanding [59]. This method is preferred in fragmented research areas involving open‐ended questions with diverse participants and where data complexity might not reach saturation for thematic analysis [60]. Elo and Kyngäs' [61] structured three‐phase approach of preparation, organisation and reporting will guide the process. First, the data will be read repeatedly to understand the context, with line‐by‐line coding and a codebook drafted for consistency and transparency [62, 63]. To enhance the credibility of coding, the bilingual researchers who conducted and translated the in‐language interviews will review the codebook [64]. The codebook will be iteratively refined, and discrepancies resolved through consensus for conformability and bias minimisation [64]. Second, similar codes will be grouped into categories, developing a coding scheme and refined hierarchical themes. Senior qualitative researchers will review methodological decisions and code interpretations to enhance dependability [65]. Third, the findings will be narratively synthesised with detailed contextual data descriptions to enhance transferability [66]. An audit trail containing original transcripts, data analysis documents and feedback notes will be compiled for further transparency [65].

Quantitative and qualitative findings will be reported separately and then converged at the interpretation stage to appreciate the initiative's effectiveness in improving participants' dementia knowledge, attitudes and supportive practices. While not as robust as triangulation, converging data is preferred for small, diverse anticipated samples and can reduce systematic biases and limitations of single‐source methods [67]. The initiative's perceived effectiveness will be interpreted through social constructivism theory to understand how participants' learning was facilitated [38].

### 5.3 Adoption by Stakeholders

After co‐delivery concludes, a semi‐structured focus group about the initiative's adoption will be held with the stakeholders involved in co‐design and co‐delivery. The discussion will examine if, how and why service providers endorse the initiative by analysing adoption barriers and facilitators in depth, capturing diverse perspectives that reflect organisational uptake of dementia supportive practices (see Supporting Information 3 for the full guide). The hybrid focus groups will be held online and in person at a local community facility to support accessibility. Participants will receive a focus group guide and information sheet via email 2 weeks prior detailing the process, risks, benefits and discussion topics. Before commencing the focus group, key details will be reiterated, including privacy, respectful behaviour and confidentiality until the project concludes. Verbal consent will be sought from participants to audio‐record the focus group. The data will be transcribed verbatim for thematic analysis using NVivo version 14. Repeated patterns of latent ideas, concepts and meaning will be identified by progressively coding from description to interpretation [68]. This rigorous approach is preferred over content analysis for the diverse, complex responses expected, allowing data‐driven insights on topics with fragmented literature, smaller samples and hard‐to‐reach populations [58, 61].

Braun and Clarke's [68] six phases of thematic analysis will guide the process. First, transcripts will be read repeatedly and coded to record initial ideas. To mitigate potential bias, a senior qualitative researcher will review preliminary codes, with discrepancies and data saturation discussed with the team. Second, broader codes will be systematically constructed, and relevant data will be arranged into categories. Phases three to five will involve reviewing, defining and labelling themes. Positionality bias and analytical decisions will be documented through reflective journaling and debriefing with a senior researcher to ensure theme clarity [66]. Lastly, transcript excerpts will be selected to illuminate findings. The final set of interpreted themes will be triangulated with the implementation findings described below.

### 5.4 Implementation by Co‐Facilitators

After co‐delivery concludes, the trained co‐facilitators will be invited to a semi‐structured focus group to discuss the initiative's implementation characteristics, fidelity, strengths and areas for improvement (see Supporting Information 4 for the full guide). The focus group guide and participant information sheet will be sent via email 2 weeks prior. Before commencing the focus group, participants will be reminded of the importance of privacy, respectful behaviour and confidentiality. With participants' verbal consent, the focus group will be audio‐recorded. The audio‐recorded file will be transcribed verbatim and analysed using the same inductive thematic analysis approach described for the adoption focus group.

The findings from the two aforesaid focus groups will be triangulated to identify the barriers and facilitators of the intervention's adoption and implementation. Flick's [69] conceptual framework will guide explicit within‐method data source triangulation. Data sources will be analysed separately to preserve each group's perspective. Comparisons will then identify convergence, divergence and complementarity across the focus group themes [69]. Insights will be synthesised to create a cohesive narrative that enhances contextual understanding and trustworthiness of findings [70]. The triangulated findings will be detailed within the context of the diffusion of innovations theory [40] to enable a comprehensive understanding of how and why the initiative was integrated within local communities by stakeholders. The intervention's adoption‐related attributes (e.g., operational ease and cost), its key enablers (i.e., stakeholders) and their decision‐making will be considered. Analysing implementation‐related characteristics like trialability (i.e., pilot opportunities) and compatibility with communities will aid understanding organisational‐level impact [41, 42].

### 5.5 Maintenance

As articulated in the effectiveness measures, the survey will be re‐administered 12 months post‐intervention to assess participants' sustained dementia knowledge and attitudes. Interviews were not feasible due to constraints in time, funding and resourcing. Survey data from the pre‐intervention (Time 1), post‐intervention (Time 2) and follow‐up (Time 3) time points will be analysed using SPSS version 29 to estimate the effectiveness of the intervention in percentage points. Homogeneity of variances, normality tests and outliers for each scale item will be assessed [57]. The pre‐intervention versus post‐intervention and post‐intervention versus follow‐up change in each DKAS and DDAS item will be assessed using independent samples *t*‐tests for normally distributed data or Mann–Whitney *U* tests for non‐normally distributed data. As survey participation could differ between time points [20, 33], group differences in demographic variables will be analysed using the *χ* ^2^ test. All outcomes will be considered statistically significant at *p* < 0.05, two‐tailed.

At the organisational level, a cost–benefit analysis will be conducted with the survey outcomes to appreciate the financial cost of maintaining the educational initiative. This is important to understand as sustainable initiatives often depend on balancing economic feasibility and ongoing community impact [33]. Acknowledging the added challenges for multicultural communities, such as barriers in languages, literacy, culture, expectations and their potential associated costs, and understanding cost–benefit dynamics are crucial [11, 13]. This analysis can inform organisational support, such as resource allocation, upscaling and ongoing delivery.

The cost of maintaining this intervention, by reusing the co‐designed programme materials, reflects the cost of service delivery. This estimate is for one in‐language delivery of the programme and includes venue hire, coordination support, facilitator time, printing of materials and catering, expressed in 2024 Australian dollars.

The benefit of maintaining the service will be estimated as the net difference in effectiveness between the post‐intervention versus pre‐intervention and the post‐intervention versus follow‐up, expressed as:

Net effectiveness=[(post‐intervention−pre‐intervention)−(post‐intervention−follow‐up)]

The adult population ( > 18 years old) of South Western Sydney in 2021 was about 359,000 [27], while the number of people living with dementia in the region was 13,457 [26]. Given that < 0.04% of children have dementia in Australia, it was assumed that all dementia cases were adults [71], so that the prevalence rate of dementia in the region is 4%. With a target sample size of 120 participants in this study, a clinically significant impact of the intervention will be observed in approximately 5 participants (4% of 120 participants). Thus, the net effectiveness of the intervention will be multiplied by 5 [5] to obtain the total number of participants who will experience a clinically significant impact of the intervention. This will be a conservative estimate as most people with dementia are aged over 65 years. The average onset age for dementia in Australia is 65 years, and the average life expectancy of people with dementia is about 10 years [26]. Another study has shown that people with dementia die 5 years earlier than people without dementia [72]. This indicates that the average benefit of the intervention will be conservatively observed within 5 years. Studies have provided willingness‐to‐pay (WTP) values to reduce carers' burden for people with dementia. One study has shown that the WTP for dementia caregiving intervention to reduce carer's burden ranges from USD $1.06 to USD $9.57 per hour [73]. Another study has shown that one additional hour of providing people with dementia assistance was associated with USD $1.64 (95% CI: USD $0.23, USD $3.04) WTP for an in‐home programme to reduce behavioural symptoms and caregiver stress [74]. The second WTP estimate, which is more conservative, will be converted to 2024 Australia dollar and applied in the cost–benefit analysis. Given that the average time spent by carer's providing care for people with dementia is 40 h per week in South‐Western Sydney [75], the WTP per hour will be multiplied by 40 h and then by 52 weeks to obtain the total WTP per annum. The WTP per annum will be multiplied by 5 years to obtain the lifetime WTP to reduce carer's burden. The lifetime WTP also represents the perceived total benefit of the intervention in reducing carer's burden due to the effect of the education programme.

The benefit–cost ratio will be determined by dividing the total perceived benefit by the initiative's total cost. This metric provides a valuable indicator of the value for money of the education programme. This analysis will be guided by a health economist specialising in dementia research.

## 6 Discussion

Dementia Friends Unite aims to improve dementia knowledge, attitudes and support practices among Arabic, Cantonese, English, Greek, Mandarin and Vietnamese‐speaking communities through an educational intervention, co‐produced with a representative stakeholder group. Using a comprehensive approach to co‐production, theory‐informed education and implementation science, this project addresses unmet community needs [3, 7, 8] and research gaps concerning best‐practice dementia education in multicultural contexts [9, 11, 13].

Co‐production has value in building capacity and integrating social groups, such as academics, service users and service providers [14, 17]. It can foster trust, empowerment, destigmatisation and democratic capital, increasing the likelihood that lessons from research will be practised [15, 21]. However, conflicting stakeholder priorities can hinder inclusivity and idea development [16, 18, 19]. In this project, stakeholders will be engaged in all project meetings and activities, with decision‐making negotiated until consensus. The ongoing PPEET evaluations of co‐production will serve to prioritise stakeholder preferences and needs for a just initiative, rather than academics either retaining decision‐making authority or prioritising community‐driven designs that might affect an intervention's reach and adoption [39, 76]. Through co‐production, this project can leverage stakeholders' capacities, networks and resources for workforces that are more responsive to community needs [11, 22].

The design of this project creates several methodological limitations that warrant mention. As this is a pilot study, the small sample size anticipated will not allow language group comparisons. Survey data are expected to be unmatched due to variable participant attendance across the intervention time points [20, 33]. This could impact the validity of the follow‐up findings. However, as a pilot study, such data can guide more rigorous, controlled trials where matched data could be obtained between time points and losses to follow‐up can be accounted for. This project was also designed according to resource constraints, thus limiting the scope of follow‐up interviews to explore longer‐term behavioural changes. The use of WTP values, from the United States, might have limited applicability in the Australian context. The lack of Australian WTP data led to the use of external WTP values. The results should therefore be interpreted with caution. This study also failed to capture the dynamics of dementia progression. Some cases of dementia may get worse, such that home caring by a caregiver may not be sustainable and would require transitioning to a nursing home.

To the best of our knowledge, Dementia Friends Unite will be the first co‐produced dementia education intervention delivered in a multicultural context. Grounded in implementation science, this multimethod project combines evidence‐based practices and theories to enhance understandings of the initiative's impact, focusing on both its design and implementation. The project could inform future community‐based research and education with multicultural populations. Importantly, the initiative addresses dementia awareness among culturally and linguistically diverse communities to promote equitable access to education and reduce health disparities [7]. Such educational and outreach efforts heed calls for public health interventions targeting awareness raising and stigma [2], including Australia's National Dementia Action Plan [77] and Culturally and Linguistically Diverse Dementia Research Action Plan [78].

## Author Contributions

**Gabriela Caballero:** conceptualisation, investigation, writing – original draft, methodology, writing – review and editing, project administration, formal analysis, software, data curation, resources. **Ann Dadich:** conceptualisation, methodology, validation, writing – review and editing, funding acquisition, project administration, supervision, resources. **Michelle DiGiacomo:** conceptualisation, methodology, project administration, resources, validation, writing – review and editing, funding acquisition. **Nicky Morrison:** conceptualisation, methodology, funding acquisition, writing – review and editing, resources, project administration. **Charles Okafor:** formal analysis, methodology, data curation, resources, writing – review and editing, writing – original draft. **Joyce Siette:** investigation, writing – review and editing, validation, project administration, data curation, supervision, resources. **Genevieve Z. Steiner‐Lim:** data curation, supervision, resources, project administration, methodology, conceptualisation, investigation, validation, writing – review and editing, funding acquisition. **Diana Karamacoska:** conceptualisation, investigation, funding acquisition, writing – review and editing, visualisation, writing – original draft, validation, methodology, software, formal analysis, project administration, data curation, supervision, resources.

## Ethics Statement

The study was approved by the Western Sydney University Human Research Ethics Committee (reference number: H15904).

## Conflicts of Interest

As a medical research institute, NICM receives research grants and donations from foundations, universities, government agencies, individuals and industry. Sponsors and donors provide untied funding for work to advance the vision and mission of the institute. The project that is the subject of this article was not undertaken as part of a contractual relationship with any organisation other than the funding declared. G.Z.S. and D.K. are employed by NICM and are members of the Canterbury‐Bankstown Alliance but have no conflicts to declare. All other authors have no conflicts to declare.

## Supporting information

Protocol for Inclusive Dementia Education Supps.

## Data Availability

The authors have nothing to report.
