# Supplementary material for: Co‐Producing and Evaluating a Culturally Inclusive Dementia Education Initiative: A Multimethod Study Protocol
Source: Health Expect. 2025 May 27;28(3):e70307. doi: 10.1111/hex.70307 (PMC12116946; doi:10.1111/hex.70307)
Supplement: Supplementary file 1 — Protocol for Inclusive Dementia Education Supps. [file HEX-28-e70307-s001.docx]

**Supplementary Materials 1**

**Background**

Facilitating co-production in dementia research can be complex in multicultural contexts due to stakeholders’ diverse perspectives and priorities (17-19). This diversity can hinder co-production integrity, fidelity, and consensus-building processes (23). This often leads to researchers inadvertently retaining authority with restricted service user involvement via consultation, rather than meaningful partnerships (17,76). Engagement efforts must acknowledge power differentials by being flexible to adapt co-production methods that meet the needs of minoritised stakeholders (18). Furthermore, the heterogeneity of co-production methods and inconsistent evaluations limit understandings of implementation determinants and scalability (15). This contributes to knowledge gaps about the implementation and impact of co-producing with and for culturally diverse populations (28-30).

**Supplementary Table S1**

*Dementia Friends Unite stakeholder groups involved in co-production*

| **Canterbury-Bankstown Dementia Alliance***  (*n* = 9) | **South Western Sydney Dementia Network**  (*n* = 8) | **Dementia Advisory Group**  (*n* = 7) |
| --- | --- | --- |
| Australian Nursing Home Foundation | Camden Council | Arabic care-partner |
| Bankstown Dementia  Carers Group | Canterbury-Bankstown Council | Chinese former care-partner |
| Canterbury-Bankstown Council | Harbison | English-speaking living experience member |
| CASS Care | Myrtle Cottage | English-speaking care partners (*n*=2) |
| HammondCare | South Western Sydney Local Health District | Greek former care-partner |
| Leigh Place | South Western Sydney Primary Health Network | Vietnamese care-partner |
| Tripoli Mena Association | Wingecarribee Shire Council | - |
| South Western Sydney Local Health District | Wollondilly Shire Council | - |
| Western Sydney University | - | - |
| *Note:* Care-partner = An informal support person for someone who lived with or is currently living with dementia; * = The Canterbury-Bankstown Dementia Alliance was an existing community group established prior to project commencement.  **Stakeholder Coordination**  Stakeholder recruitment targeted local multicultural service users and providers, interested in improving dementia understanding in their respective communities. Guided by previous research (15,23,39), the recruitment processes aimed to promote accurate community representation and alignment of priorities (see Supplementary Table S2).  Stakeholders entrusted the research team as project collaborators and facilitators to leverage their academic and project management expertise. This decision aligns with recommendations for engaging vulnerable and minoritised populations in participatory research (36). This differs from other co-production methods that can be less impactful by relying exclusively on stakeholder input or where academics retain authority (37). As decided with the stakeholders, the research team’s responsibilities will include managing email correspondence, taking meeting minutes, mediating conflicts, incorporating diverse perspectives and theoretical principles, and drafting educational resources for refinement during co-design workshops (23). All project-related materials will be sent at least seven days in advance for independent review, with the option of providing feedback via email or telephone. | | |

**Supplementary Table S2**

*Recommendations implemented for the recruitment, collaboration, and support of the Dementia Friends Unite stakeholders*

|  | **Recommendation(s)** | **Author(s)** |
| --- | --- | --- |
| Recruitment | Recruit through intermediaries | Hurley et al., (2021) |
|  | Recruit both people with lived and caring experiences | Goh et al., (2022) & Wang et al., (2019) |
|  | Recruit people who know each other | Wang et al., (2019) |
|  | Use local outreach methods to build trust, raise awareness, and engage people in dementia research | Brijnath et al., (2022) |
|  | Use materials that consider differences in education and dementia literacy |  |
| Collaborative processes | Prioritise storytelling and an open dialogue approach | Goh et al., (2022) & Wang et al., (2019) |
|  | Recognise cultural differences and diversity within ethnic minority populations | Brijnath et al., (2022) |
|  | Involve bilingual/bicultural workers in multiple project phases (e.g., data collection, analysis, dissemination) |  |
|  | Researchers serve as project managers (e.g., organising meetings and stakeholder communication) | Hurley et al., (2021) |
|  | Researchers serve as facilitators (e.g., guiding co-design workshops, integrating research and theory into design) |  |
|  | Researchers must remain flexible | Wang et al., (2019) |
|  | People with lived and caring experiences share their opinions independently |  |
|  | Clear communication about the purpose of tasks |  |
|  | Schedule time for breaks |  |
|  | Use topics to guide structured discussion sessions |  |
|  | Organise smaller groups for discussion |  |
|  | Recap previous sessions |  |
|  | Compartmentalise main tasks into subtasks |  |
|  | Use tangible and culture-appropriate materials |  |
|  | Clarify needs and capacity prior to tasks (e.g., eyesight, memory, hearing) |  |
|  | Caregivers assist people living with dementia during activities, facilitating communication with researchers and others in the group |  |
| Participation support | Monetary incentives^a^ | Hurley et al., (2021) & Wang et al., (2019) |
|  | Organise travel to meeting locations | Hurley et al., (2021) |
|  | Offer accessible hybrid meetings | Wang et al., (2019) |
|  | Dedicated research participation support person | Goh et al., (2022) |
| *Note:* All recommendations will be implemented to promote equitable stakeholder participation.  ^a^As this project is anticipated to run for three years, stakeholders were asked to attend monthly hybrid meetings in the first year and quarterly meetings for the last two years. The service providers had the associated project workload integrated into their existing organisational roles, providing in-kind contributions of salaries and time. To ensure equitable participation for Dementia Advisory Group members, $50 gift cards were made available per hour spent on project activities. A research participation support person will facilitate regular communications and transport options for members with living and caring experiences throughout the project lifecycle. | | |

**Supplementary Materials 2**

**Participant Interview Guide** – Conducted one-month post-intervention.

**Interviewer instructions:** Begin the interview with an introduction about yourself, your role on the project, and the purpose of the interview.

**Suggested explanation of interview purpose:** Since you participated in the dementia education workshops a month ago, we would like to understand what you thought about these.  We will also ask you questions about your age, gender, country of birth, etc. You do not have to answer all the questions if you do not wish to. The interview will take about 30 minutes to complete. You can take breaks if you feel the need. You do not have to participate in this if you do not wish to and you can stop at any time.

Do you have any questions for me?

When you’re ready, I will press record and we can begin the interview with your verbal consent.

**Obtain verbal consent to participation:** After pressing the record button, please advise the participant to provide verbal consent to confirm their agreeance to participate in the interview.

**Part One – Demographics**

In this first section, I am going to ask you a bit about yourself.

- What is your age?
- Which gender do you identify as?
  - Man or male
  - Woman or female
  - Non-binary
  - I use a different term (please specify): ___________
  - Prefer not to say
- Which country were you born in?
- Which languages do you speak at home?
- Which suburb do you currently reside in? (name or postcode)
- What is your employment status? (Unemployed/retired; volunteering; working)
- What is the highest level of education that you’ve achieved?
- Do you have any personal experience working with people living with dementia or carers?
  - If yes, can you please elaborate on this experience?
- Do you have any professional experience working with people living with dementia or carers?
  - If yes, can you please elaborate on this experience?
- Have you ever had any training on dementia?
  - If yes, can you recall when this training was and what was the focus?

**Part Two – Program Perceptions**

I’m now going to ask about your perceptions of the workshops.

- Can you tell me why you decided to attend the dementia education workshops?
- How many sessions did you attend? Which ones were they? *(Prompt any of the names, dates, and locations of the workshops they attended in their language)*
- How has the program impacted you, if at all?  *(Prompts: has it changed your thoughts, attitudes, understanding, behaviours, interactions towards people living with dementia e.g.,*
  - Has *your perception of dementia changed? If so, how?*
  - Are *you more understanding to people in your life who are impacted by dementia?*
  - Have *you modified your language or behaviour to communicate more easily with a person with dementia?*
  - Did *you make any changes around your place to make it safer and easier for people with dementia to access?*
  - Have *you sought help from any of the services we talked about, like Dementia Australia or My Aged Care?)*
- What, if anything, has stopped you from putting the knowledge from these workshops to use in your day-to-day life?
- Do you think participating in this program and applying what you’ve learned in your everyday life can help improve the care provided for people living with dementia? Why and/or in what way?
- What did you think of the duration of these workshops? *(Prompt: was it too long, too short?)* Should it be changed (If yes, in what way)?
- Would you recommend this program to someone? Why/why not?
- What areas could we improve on? *(Prompt: were there any topics you want to know more about?)*
- Is there anything else you want to say about the workshops?

**Interview Conclusion**

**Interviewer instructions:** Thank the interviewee for their time and contributions. Explain that their interview data will be deidentified and transcribed for analysis. We will aim to share the findings in 2024. If they have any further questions, they can contact the project leader Dr Diana Karamacoska on 0479 150 816.

**Supplementary Materials 3**

**Adoption Focus Group Guide** – Conducted post-intervention to assess adoption by stakeholders.

**Interviewer instructions:** Begin the interview with a brief welcome to all the stakeholders and the purpose of the focus group.

**Suggested explanation of interview purpose:** Today’s audio-recorded focus group serves to evaluate your perspective about the dementia workshops that we ran in your community. Please avoid using names and passing judgements during this session. Be respectful and maintain confidentiality in and outside this session. If you’re unsure of anything, please ask your facilitator any questions you may have.

Do you have any questions for me?

When ready, express your verbal consent to being included in this research. I will press record to begin the focus group and step through each question below. Take your time to reflect on the question before speaking. Remember: you do not have to participate in this if you do not want to.

**Questions For All Stakeholders**

1. Why did you or your organisation decide to be involved in this initiative?
2. What helped make this initiative successful?
3. What were the barriers to its success?
4. Will you or your organisation want to continue running this initiative in the future? If so, what will help you do this? If not, why?

**Supplementary Materials 4**

**Implementation Focus Group Guide** – Conducted post-intervention to assess implementation by co-facilitators.

**Interviewer instructions:** Begin the interview with an introduction about yourself, your role on the project, and the purpose of the focus group.

**Suggested explanation of interview purpose:** Today’s audio-recorded focus group serves to evaluate the co-facilitation of dementia workshops in your community. Please avoid using names and passing judgements during this session. Be respectful and maintain confidentiality in and outside this session. If you’re unsure of anything, please ask your facilitator any questions you may have.

Do you have any questions for me?

When ready, express your verbal consent to being included in this research. I will press record to begin the focus group and step through each question below. Take your time to reflect on the question before speaking. Remember: you do not have to participate in this if you do not want to.

**Questions** **For Co-Facilitators**

- How effective was this program in raising awareness about memory loss and dementia for yourself and the participants that attended the session?
- Was the program effective in changing the beliefs and attitudes about dementia amongst participants and yourselves? Why or why not?
- How effective was this program in appreciating cultural perceptions of dementia?
- What were your experiences of the in-person deliveries of the program? What were the strengths and weaknesses of these?
  - *Prompts: content, design, delivery, community-based facilities/locations, how did this enhance/hinder interaction among participants and between yourself and the participants?*
- How did you perceive the co-facilitation process? What were the strengths and weaknesses of this?
  - *Prompts: WSU staff support and collaboration, how did this approach enhance or hinder participant responsivity and engagement?*
- How sustainable is a program that operates with co-facilitators?
- Did you need to alter any of the content to convey a point? If so, what was it and how did you do it?
  - *Prompts: adherence to the material; use of facilitator notes; including real-life examples; activities and discussions; how appropriate was the content written on the slides and did you have to modify this? Did you skip any material?*
- How could this program be improved?
- Is there anything else that you’d like to share or discuss that we haven’t covered?
